# Development of the Rational Thinking, Emotion Regulation, and Problem-Solving Mental Fitness Mobile App for US Navy Sailors: Qualitative and Quantitative Usability Evaluation

**DOI:** 10.2196/89994

**Published:** 2026-07-31

**Authors:** David P Cenkner, Jennifer June, Stephen M Schueller, Alyson K Zalta, Joseph Grammer, Eric Ekman, Kailee Baldini, Jessica M LaCroix, Marjan Ghahramanlou-Holloway

**Affiliations:** 1 Department of Psychology, School of Social Ecology, University of California, 214 Pereira Dr, Irvine, CA, 92617, United States, 1 724-787-0991; 2 The Informatics Applications Group, Reston, VA, United States; 3 Department of Medical and Clinical Psychology, Uniformed Services University of the Health Sciences, Bethesda, MD, United States; 4 Department of Psychiatry, Uniformed Services University of the Health Sciences, Bethesda, MD, United States

**Keywords:** human-centered design, digital mental health, mental fitness, suicide prevention, military, usability testing, military mental health, mobile intervention, early career, service members, mobile health, mHealth

## Abstract

**Background:**

US Navy sailors experience a range of personal and interpersonal stressors due to challenging operational tempos that can contribute to psychological distress and increased suicide risk. Digital mental fitness tools offer scalable, stigma-reducing solutions to support psychological readiness. However, many existing digital mental health interventions use top-down designs that lack input from the target population and focus on treating psychological symptoms rather than improving overall mental fitness.

**Objective:**

This study aimed to design a mobile app version of the Rational Thinking, Emotion Regulation, and Problem Solving (REPS) program and evaluate usability and user experience. REPS is grounded in cognitive-behavioral and rational emotive behavior therapy principles. We examined sailor feedback across two evaluation phases to make iterative improvements to the app.

**Methods:**

Two rounds of usability testing were conducted with 13 first-term Navy sailors over a 2-month period in spring of 2024. Sailors on duty at a naval ship were recruited by their commanding officer. Evaluation phase 1 (n=8) included exploratory interviews and concept testing using an initial prototype that covered a learning objective of the first module. Sailor feedback from phase 1 informed iterative refinements, including a gamified badge feature and dedicated skill practice session. Evaluation phase 2 (n=5) included a think-aloud protocol using an updated, fully functioning prototype of the first module, with three structured learning objectives. Both phases assessed sailor feedback through qualitative interviews and the System Usability Scale (SUS). Thematic analysis identified core themes related to user needs and app characteristics, and SUS scores were summarized to describe usability.

**Results:**

Sailors found the REPS app prototype to be culturally relevant and relatable to their daily life within the Navy. Sailors also commented that the app had an intuitive, user-friendly design and praised the modern and visually appealing interface. Sailors reported liking the scenario-based situations and clear explanation of how thoughts, feelings, and actions are intertwined. Sailors also identified ways to enhance user engagement, including clearly defining psychological terminology, introducing gamified rewards, outlining learning objectives, and incorporating more prominent visual cues to enhance the discoverability of interactive content. Quantitatively, SUS scores for both evaluation phases were in the excellent range (phase 1: median 92.50, range 72.5‐95.0; phase 2: median 85.00, range 80.0‐92.5).

**Conclusions:**

The REPS mobile app was rated as highly relevant and usable by newly enlisted Navy sailors. Scenario-based activities, intuitive design, and relatability with lived military experiences were identified as core strengths. Iterative feedback emphasized the importance of human-centered design in refining app functionality, esthetics, and information. These findings support REPS as a promising, scalable digital tool to enhance mental fitness and contribute to universal suicide prevention in the Navy.

## Introduction

Improved psychological health of active-duty US military service members is critical for optimized performance, protection against mental health conditions, including suicidal thoughts and behaviors, overall mission readiness, and unit cohesion. Notably, mental health diagnoses have increased from 2021 to 2023 among US service members, with depressive disorders ranking among one of the most frequent [1]. Furthermore, suicide is consistently noted as one of the three most common causes of death for active-duty US service members [2]. Research has found an increase in suicide risk during the initial months of military service, in which newly enlisted service members are faced with novel mental and physical challenges [3]. Underdeveloped mental fitness skills can contribute to increased stress, deterioration of effective coping, and reduced readiness, potentially leading to an increase in suicide risk [4,5]. Given the recent recommendations from the Defense Health Board to move toward an institutional model of resilience [6], efforts to target mental fitness early in training among newly enlisted US service members are needed to improve psychological health and reduce reliance on harmful coping strategies such as nonsuicidal and suicidal self-directed violence [3].

Although service members benefit from participating in mental health programs and treatments that improve psychological health, several barriers may prevent their willingness to engage. For example, service members may have concerns over stigma, career impact, and may hold negative attitudes toward mental health treatment that may prevent them from seeking care [7]. Many service members have also expressed a desire to handle problems on their own [8]. Thus, easily accessible and anonymous mental health interventions can reduce barriers and facilitators to care and improve suicide prevention among service members.

Digital mental health interventions (DMHIs) may be one way to reduce barriers to traditional mental health care. DMHIs are programs delivered via websites or mobile apps aimed to maintain and improve emotional and cognitive well-being to prevent psychological distress and enhance skills in managing life stressors and the evolving demands of a military career. Among DMHIs, apps that focus on mental fitness have become increasingly popular. Within mental fitness apps, individuals are taught how to improve their emotion regulation and mental resilience. Mental fitness apps have been found to decrease suicide risk and stress [9,10]. Further, mental fitness apps may address service member concerns around stigma and preference for self-reliance due to the ability to privately use the app at their convenience through their smartphone. The US Department of Veterans Affairs (VA) and Department of Defense (DoD) have developed a number of apps for veterans and service members that fall broadly into two categories: self-management apps (ie, self-help interventions used by patients independently) or treatment companion apps (ie, apps designed to be used alongside a clinician-facilitated evidence-based treatment) [11]. Most VA and DoD apps have been designed for individuals with a clinical issue, such as posttraumatic stress disorder or insomnia. Thus, apps aimed at enhancing the overall mental fitness of service members and veterans could be more widely applicable and have important benefits across the entire military population.

DMHIs face many challenges. For example, prior research has identified high user dropout and low user engagement [12,13]. This might be even more common in mental health fitness apps: users who do not have an identified mental health condition may have less motivation or perceived need to improve their mental fitness. One solution to improve user experience is early input in the app design process from members of the target population. Many existing DMHIs have used top-down approaches, digitizing existing treatments without input from intended users [14]. Thus, bottom-up approaches in app design that collaborate with the target audience may be needed to increase user acceptance and engagement.

Human-centered design (HCD) is one such bottom-up approach. HCD focuses on the user’s culture, input, and feedback in the development of the app, leading to a more accessible and usable design and product for potential stakeholders [15]. HCD typically involves collecting this information from users up front with final design decisions made by an independent design team [15]. However, few DMHIs have been found to involve users in the design process, and many HCD methods have not been fully used in digital interventions for mental health [16]. Focusing on the user may lead to increased user engagement and usage because the app may be better equipped to meet the needs and interests of the target population [17]. For example, prior review literature has found that user perceived fit, defined as the extent to which users felt the intervention targeted individuals like themselves and included relevant material to the users’ culture and values, was found to be an important facilitator to increasing user engagement [18]. Indeed, recent advances in suicide prevention digital apps have used HCD approaches to incorporate stakeholder input in designing the app to improve user engagement and acceptability [19,20]. Although prior research has shown promise in increasing user engagement and acceptability using HCD, little work has addressed this need for an active-duty service member population.

Navy sailors have a high rate of death by suicide, 21 per 100,000, per the latest available data [21]. The US Navy 2023 Health of the Force Report highlights a steady rise in stress levels among junior enlisted sailors (military rank E-6 and below; US military enlisted pay grades range from E-1, the most junior, to E-9, the most senior), with 41% reporting severe or extreme stress in 2023, a notable increase from 29% in 2019 [22]. Navy sailors who experience high job demands, long durations at sea, social isolation, poor social support, low team cohesion, and long working hours may be at increased risk for suicide [23]. Given that the majority of available DMHIs target specific clinical problems [24], developing transdiagnostic programs that target overall mental fitness has the potential for broad appeal and impact. Currently, no mobile apps are designed for service members that use mental fitness as a universal suicide prevention approach.

Rational Thinking, Emotion Regulation, and Problem Solving (REPS) is a mental fitness program for sailors that uses cognitive-behavioral and rational emotive behavior therapy (REBT) principles to improve psychological readiness and prevent suicide. Although there is uncertainty surrounding how findings from in-person content translate to digital content, testing of the in-person REPS program supported the program’s feasibility and acceptability [25]. However, it is unclear how such characteristics would carry over to a digital version of the program. This study used an HCD approach to develop a mobile app version of REPS and evaluate its usability across two phases of testing with US active-duty sailors. Specifically, we examined sailor feedback related to user needs, perceptions of app functionality, esthetics, and content, and assessed initial usability of early-stage prototypes.

## Methods

### Ethical Considerations

This usability evaluation was a quality improvement project conducted to guide the design of the mobile app. Therefore, the usability evaluation did not meet the definition of research and institutional review board approval was not required. Sailor recruitment was led by commanding officers who required volunteers for the evaluation. Once volunteers were identified, usability specialists explained the study procedures to each sailor and obtained their verbal consent to record the session.

### Design Process

#### The Initial REPS Program

The REPS program has been designed as a face-to-face, classroom-based course to be delivered to newly enlisted Navy sailors by Navy instructors. The program was developed by a collaborative, multidisciplinary team of civilian and Navy psychologists, and military culture subject matter experts at the Uniformed Services University. REPS is built on 50 years of empirical evidence supporting the efficacy of cognitive-behavioral therapy and REBT to enhance rational thinking, emotion regulation, and problem-solving skills to prevent suicide [26,27]. REPS consists of 4 modules: life changes, rational thinking, emotion regulation, and problem solving. REPS emphasizes the idea that mental fitness, like physical fitness, must be practiced regularly to adapt to change and maintain health [28].

#### Design of the REPS App

The design of the REPS app focused on two aspects. First, adaptation of the content of the REPS program was performed to make the content appropriate for mobile, self-guided delivery. Second, the development of mobile features occurred to support user comprehension and engagement. Development was supported by an interdisciplinary partnership consisting of the initial developers of the REPS program, a design team experienced in translating mental health content into digital formats for the VA, and a university-based team with experts in military trauma and DMHI development, evaluation, and implementation. Several team members had direct military service experience, enhancing the relevance and applicability of the adaptation. Adaptation of the content from the in-person REPS program began with a two-day training session led by the design team. During the training sessions, each proposed module was covered as to how it would be presented to sailors. Following the training, the design team defined the overall structure of the app and categorized the content into three key areas: (1) the explanatory material for concept instruction, (2) content that should be emphasized to increase user learning, and (3) interactive components designed to engage learners through activities. During the development of the app, team members met virtually every two weeks to draft and revise content, provide progress updates, review visual design elements, integrate user feedback, and address and resolve emerging issues.

The REPS in-person content was used as an outline for the structure and information flow of the mobile app. Prototyping was used to help visualize and test ideas, identify potential issues, gather user feedback, and refine solutions before investing in full-scale development. Figma design software was used to allow real-time collaboration between team members. Mock-ups, wireframes, and interactive prototypes were created to flesh out the learning structure, navigation flow, content and visual design, and interactive elements. Visually, the REPS app was designed to enhance user learning and retention through the use of infographics, diagrams, and illustrations that break down complex concepts into digestible segments. High-contrast color schemes and accessible font choices were used to improve readability. Further, learning objectives were highlighted through animations and transitions.

When designing the REPS app, a feature analysis of selected commercially available mental health apps was conducted to evaluate the strengths and weaknesses of their functionalities to enhance user engagement. This analysis concentrated on mental health–based learning, microlearning, and gamified activities. Mental health–based learning included scenario-based learning objectives and prompts that guide users through reflective and decision-making exercises. These exercises were created to be culturally relevant for REPS users by presenting scenario-based learning modules that reflect military stressors related to typical personal and professional experiences that sailors encounter. Microlearning (ie, breaking down content into concise, focused segments with goal-oriented learning objectives) was implemented to allow sailors to engage with learning materials during their limited free time. Research has found several benefits to microlearning, such as high acceptance, increased engagement, enhanced learning outcomes, and increased perceived confidence [29,30]. Alongside microlearning, the use of gamification elements, such as challenges, rewards, and progress tracking, was incorporated into the REPS app design to improve user engagement, retention, and motivation. For example, users of the REPS app can earn medals and achievement badges as they progress through the app. One prior study found significant improvements in both retention and engagement for students using a gamified app compared to students not using a gamified app [31]. Finally, REPS follows the “Tell, Show, Do, and Review” approach: introduce key concepts and objectives of the module, present examples of the concepts, provide interactive exercises for practice and skill reinforcement, and provide opportunities to reflect on content to solidify learning outcomes. Prior research has found that active engagement with learning materials is associated with behavioral change [32,33].

**Figure 1.**
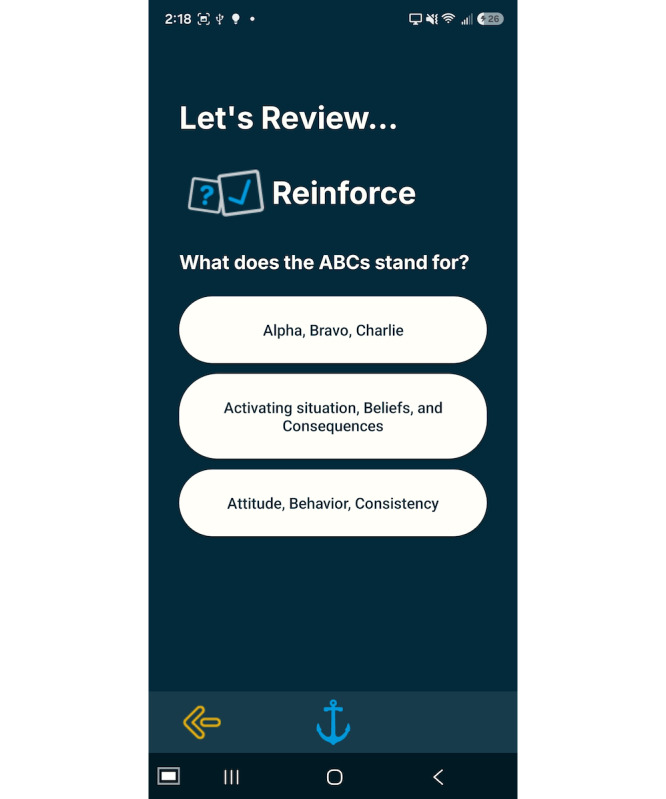
Screenshot of the REPS (Rational Thinking, Emotion Regulation, and Problem Solving) app displaying a question at the end of a learning objective to reinforce concepts that were taught within the module.

**Figure 2.**
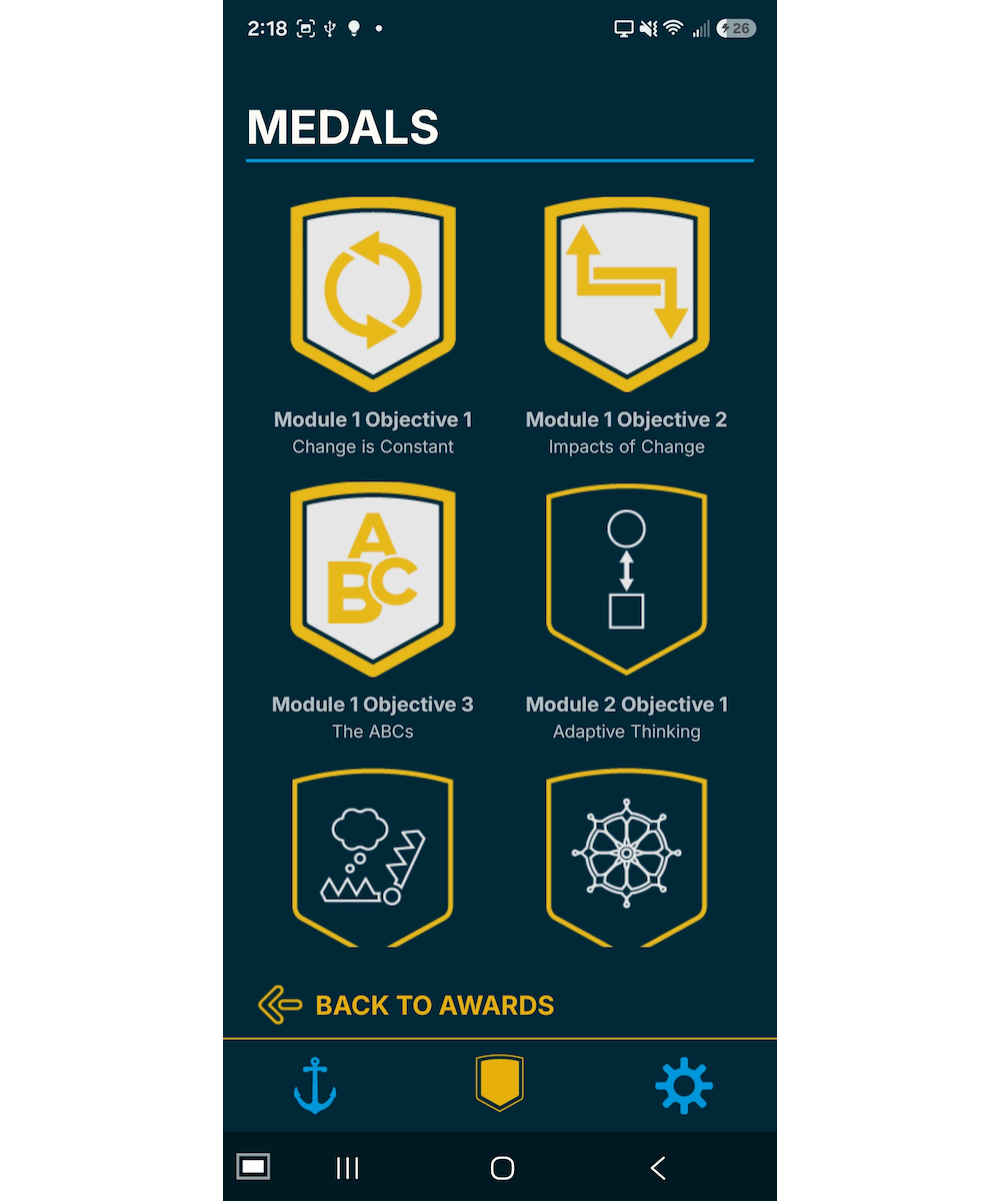
Screenshot of the REPS (Rational Thinking, Emotion Regulation, and Problem Solving) app displaying some of the medals that participants can earn when completing tasks within the learning module. The home screen button within the REPS app is represented by an anchor to tailor the app to the Navy population.

The REPS app was developed for both iOS (Apple Inc.) and Android (Google LLC) systems. Further, consideration was given to the logistical and technical limitations that might impact sailors’ use of their mobile devices during deployment. For example, it was critical to develop the REPS app to work fully offline to assist sailors who are underway on a ship, stationed abroad, or in isolated locations with poor internet connectivity. These offline options guarantee that sailors can reliably access the REPS app, regardless of location or connectivity challenges. See Figures 1 and 2 for screenshots of the REPS app.

### Procedures

#### Overview

The primary objectives of the two evaluation phases were to gather information about user needs, app functionality, app esthetics, app information, and app usability. These evaluation phases took place on a naval ship and were conducted during a two-month period, approximately seven weeks apart during the spring of 2024. Sailors were recruited by their commanding officer and were invited to participate on a volunteer basis. A total of 13 sailors were recruited across both phases, reflecting the available pool of volunteers at the single naval ship site and is reflective of sample sizes reported in comparable HCD usability studies [19,20]. All sessions were conducted one-on-one with one of two usability specialists, lasted between 45 and 60 minutes, and depending on the evaluation phase (detailed below) included collecting demographic information, a brief presession interview, task-based interactions with a prototype, postsession questions, and collecting responses to the System Usability Scale (SUS) [34]. The SUS is a 10-item self-report questionnaire to assess technology for usability and learnability. Sailors were asked to rate their experience with the REPS content on a 5-point scale from 0 (strongly disagree) to 4 (strongly agree). Calculation of the total SUS score is completed by summing items and then multiplying by 2.5. Therefore, scores on the SUS can range from 0 to 100. All testing was conducted using a prototype on an Apple iPhone 12. The phase 1 and phase 2 interview guides can be found in Multimedia Appendix 1 and Multimedia Appendix 2.

#### Phase 1

The primary goal of phase 1 was to gather broad exploratory feedback on app content, design, and navigation from newly enlisted sailors. During the first phase, eight sailors engaged in an exploratory interview and reviewed a prototype of the initial design for the REPS mobile app. The interview focused on sailors’ experiences with stress management, maintaining mental fitness in the Navy, and using mobile or digital tools for training and self-improvement. Afterward, sailors interacted with an interactive prototype that included the first learning objective in Module 1, as well as several design concepts for interactive learning exercises. The prototype showed key navigation elements, feedback features, and sample content aimed at testing the instructional tone and overall design flow. Once they completed the walkthrough, sailors took part in a short debrief interview and filled out the SUS to provide preliminary feedback on the app’s design, usability, and overall functionality. Prototype refinements between phases were informed by sailor feedback from phase one and discussed collaboratively during team meetings.

#### Phase 2

The primary goal of phase 2 was to evaluate usability and user experience using a more complete and structured prototype. Informed by phase 1 findings, the prototype was updated to include a choose-your-own-adventure activity format, a gamified badge collection feature, a dedicated skill practice section (“Do Your REPS”), and explicitly defined words that were unfamiliar to sailors during phase one (eg, maladaptive). For the second phase, five sailors took part in usability sessions using a think-aloud protocol while navigating the updated prototype of the REPS app. These sailors completed a brief onboarding, explored the home screen, and worked through Module 1, which included three structured learning objectives. Sailors were encouraged to verbalize their thoughts, reactions, and decision-making processes as they interacted with each section of Module 1. After each learning objective, sailors provided qualitative feedback on usability, content clarity, and engagement, followed by a short debrief and completion of the SUS to assess the app’s usability and overall user experience across iterations.

### Data Analytic Approach

A thematic analysis was performed to identify key patterns and insights from sailors’ feedback and observations gathered by usability specialists across both usability phases. Thematic analysis was conducted after the completion of both evaluation phases to allow for coherent cross-phase synthesis within the Mobile Application Rating Scale (MARS) framework [35], which includes dimensions of functionality, esthetics, engagement, and information, providing a theoretically grounded structure for organizing findings. Iterative prototype refinements between phases were informed by sailor feedback discussed collaboratively during team meetings rather than a formal analysis. Within each MARS category, the lead usability specialist reviewed all interview transcripts and observation notes to develop initial codes representing user needs, perceptions, and usability issues [36,37]. The data were then organized through affinity diagramming, where related ideas were grouped into clusters to reveal detailed descriptive patterns and broader conceptual themes [38,39]. This iterative process allowed the team to synthesize individual user comments into higher-level insights about the REPS app’s usability and learning design, while maintaining alignment with an established framework for mobile app evaluation. Recommendations from the study team, guided by user input, are presented in the following section.

The overall mean, SD, median, and range of the SUS and the usability and learnability subscales were calculated. Interpretive guidelines of mean SUS scores based on normative data found that a score of less than 20.3 is considered awful*,* 20.4‐35.7 is poor, 35.8‐50.9 is okay, 51.0‐71.4 is good, 71.5‐85.5 is excellent, and anything greater than 85.6 is the best imaginable [40].

## Results

### Users

Thirteen first-term Navy sailors engaged with our team across the two evaluation phases. The first phase included eight sailors (4/8 male, 50%) with a mean age of 23.38 (SD 3.20) years. Six (6/8, 75%) reported being deployed, and the military rank among sailors ranged from E-2 to E-5. The second phase included five sailors (4/5 male, 80%) who had a mean age of 19.8 (SD 0.45) years. No sailor in the second study reported being deployed, and the military rank among sailors ranged from E-1 to E-3. We did not collect race or ethnicity information about the sailors.

### User Needs

#### Responses

During the initial interview, sailors highlighted the unique challenges posed by deployments and long periods away from family, noting the significant toll these factors can take on their mental health. For example, one sailor reported,

Mental fitness is essential because sailors go through a lot. Specifically, not right now since we’re in the yards, so people are able to be with their families. But once you’re deployed or at a port and you’re doing small deployments, it can take a toll on people, especially sailors who have auto ship to ship.

[P6]

Another sailor reported,

I think being mentally resilient is important to handle all the challenges we face on board the ship, especially when schedules are unpredictable.

[P2]

During testing of the app, numerous sailors indicated that they found the mobile app concept to be helpful. A key theme was that sailors valued exploring different scenarios and alternative responses to understand how various decisions could play out in different contexts or situations. Sailors also highlighted the value of viewing alternative outcomes and suggested incorporating a feature to go back and review each response. For example, one sailor stated,

Instead of offering just one solution, present different versions of that solution

[P4]

Others stated,

I just wanted to see the other options so if you got something wrong, what would it look like?

[P3]

and

Is there a way to go back and see each response?

[P5]

#### Recommendations

Sailors wanted to explore and learn from various responses. Therefore, a choose-your-own-adventure style was adopted to enhance user engagement.

### App Functionality

#### Responses

Sailors appreciated the intuitive and user-friendly design of the app, which contrasted with their previous experiences with other military apps. One sailor stated,

It’s simple and to me, it seems like a good first step.

[P4]

Another sailor reported,

I think it’s easy to follow and I like the setup because most of the time on military apps, the setup is old looking and [you] can’t click any buttons. It’s just hard to follow. I think it’s very modern.

[P5]

Another sailor indicated a clear understanding of the module structure and easily navigated between screens by stating, “The app was easy to navigate through” (P9).

Four sailors viewed the app’s gamification elements, specifically badges, as generally positive and engaging features. Several sailors noted that these elements provided motivation and a sense of accomplishment as they progressed through the training. For example, one sailor stated, “The medals are a nice touch; they make you feel like you’re making progress” (P12), while another sailor reported,

I think it’s pretty good…like I said, this app kind of reminds me of Duolingo a little bit and that’s what they would do. So, I mean, it just keeps it like more engaging.

[P10]

Sailors indicated a need for greater clarity in some of the app’s activities and results pages. For example, one sailor stated,

Maybe if you could click on it, it would have a little text box that shows this is incorrect because of the connotation.

[P5]

Sailors also emphasized the importance of the app having offline capability, citing the limited internet connectivity while on deployment or at sea. One sailor reported the following:

I know we didn’t have Wi-Fi or internet, so we couldn’t use our phone. I feel like it’d probably be helpful to be able to download the app.

[P7]

#### Recommendations

User feedback highlighted several considerations for app functionality that may be important for digital mental health tools in military settings. First, incorporating gamified features, such as reward systems, can enhance user engagement by reinforcing continued use. Second, activities that emphasize constructive feedback, rather than marking responses as simply correct or incorrect, may shift the focus from judgment to learning, thereby supporting users in grasping the objectives and purpose of the activity. Third, ensuring full offline functionality is critical for tools intended for Navy sailors and similar populations who may face limited or inconsistent internet access during deployment.

### App Esthetics

#### Responses

The app’s modern and visually appealing interface also received praise. For example, one sailor reported, “I like that it is very clean and pretty to look at” (P3). Several sailors highlighted the app’s visual and interactive features as a strength that helped reinforce learning, with one sailor stating, “The images were captivating” (P12). Another sailor noted the following:

I think what stood out to me is what I just did, the triangles.…I like reading the different scenarios…like the different actions or what would happen, you know, with a person what they feel because everybody feels different.

[P10]

Two sailors expressed a desire to see an outline of the training program. They mentioned the current interface did not provide sufficient information to help them understand what the training entailed. One sailor noted,

Just give us a brief synopsis of the page or at least the few buzzwords.

[P12]

Another stated,

I would have liked a table of contents or something that gives us an overview of the app.

[P10]

Notably, the prototype included strategies to enhance user engagement within the app. For example, key vocabulary terms were presented as clickable headers, key words were highlighted with an [i] symbol next to them to indicate that additional information was available for exploration, and clickable knowledge nuggets were placed within objectives to enhance comprehension. The intent was that sailors would click these items and a pop-up containing additional information would appear. However, no sailor noticed or interacted with the clickable headers, highlighted key words, or knowledge nuggets. Furthermore, a gamified badge feature was introduced to motivate sailors to complete learning objectives. However, all sailors navigated to the wrong icon, suggesting that the design of the badge icon did not align with user expectations. Finally, during the evaluation phase, sailors tested a choose-your-own-adventure style activity that guided sailors through scenarios involving an activating event, followed by a sequence of thoughts, beliefs, and actions. Different pathways highlighted the potential consequences of decisions, ranging from helpful to harmful. Users were given the option to replay the activity and explore alternative scenarios.

However, no sailor clicked the Play Again button. One sailor did favorably view the option, stating the following:

There were ways I could have gone ahead and seen the maladaptive and adaptive way of thinking. Being able to see both sides is really nice. It helps provide context for however you might think in the future.

[P12]

#### Recommendations

Based on user feedback, several insights emerged regarding app esthetics that may inform the design of digital mental health tools for service members. First, users emphasized the importance of having a clear outline of learning material. Presenting a navigable overview of modules and learning objectives may help users orient themselves within the training program. Second, discoverability and engagement appear to benefit from salient visual cues (eg, animations or callouts) that draw attention to key material. Third, the cultural relevance of icons and symbols (eg, using an anchor to represent the Home button) may enhance resonance with the target population. Finally, providing contextual prompts that clarify the purpose and outcomes of specific features (eg, replay options in interactive activities) may encourage continued use and deeper engagement.

### App Information

#### Responses

The app’s information resonated strongly with sailors, who appreciated its alignment with their daily challenges and lived experiences in the Navy. For example, one sailor stated,

I like that it’s relatable and for people who have been on other ships or even gotten underway, it kind of hits home for them.

[P4]

Another sailor stated, “It’s really relatable to most sailors’ day-to-day challenges” (P8). A different sailor also noted,

The scenarios were relatable, down-to-earth, and it really could be anybody in those situations, which made it easier to connect with.

[P12]

Further, the app’s scenarios and interactive elements were engaging and relatable, resonating with users’ experiences. One sailor noted,

What stood out to me was how they explained the ABCs because I think they are really important to know how you react and what you think in a situation because it can really affect the outcome.

[P10]

Sailors were also directed to go to the “Do Your REPS” section of the prototype and give their thoughts about practicing the techniques and activities they learned in Module 1. The feedback from four sailors was positive. One sailor specifically mentioned the importance of learning how to react to different situations by stating,

“I would say it would give you a lot of thoughts and ideas on how to handle situations in the future.

[P10]

Three sailors specifically commented on vocabulary words and keywords on one of the prototype screens. For example, one stated,

So like the thinking traps, I think this is a good way to kind of show what the other responses are.

[P5]

Another sailor stated,

Everyone has unhelpful thoughts at times, but the key is recognizing when you fall into a thinking trap so you can better manage your thoughts and reactions.

[P6]

Another sailor stated,

It was easy to follow along and remember because they used keywords and buzzwords that I feel would definitely stick with people.

[P12]

Other sailors expressed unfamiliarity and curiosity about terms like maladaptive, highlighting the importance of clarity when introducing psychological terminology. For example, one sailor reported, “I never heard that word before [maladaptive] so then I kind of put that into like a different perspective” (P3), while another stated, “I was very confused at first and it took a little bit of elaborating for me to understand” (P2).

One dislike that emerged was that the app was too simplistic. One sailor reported that he felt the learning was a bit childlike and thought it needed to be more challenging. This sailor reported,

I mean, the only thing I would say I disliked is it’s a personal thing. It just it felt…simplistic. Because I feel like this is a sort of app I could see in a high school or elementary school and we’re doing it as grown adults, we can handle some tough reading.

[P12]

#### Recommendations

User feedback also pointed to considerations for improving the clarity and delivery of information within digital mental health tools. First, defining key terms in simple, relatable language before introducing them in activities may support comprehension. Concrete examples or relatable quotes can further illustrate complex concepts, such as distinguishing adaptive from maladaptive behaviors. Second, apps in this space need to balance simplicity and accessibility with sufficient depth to apply to the target population. Sailors emphasized a preference for materials that engaged them as adults, suggesting that effective design may combine straightforward functionality and esthetics with content that conveys maturity and respect for the user’s lived experience.

### App Usability

**Figure 3.**
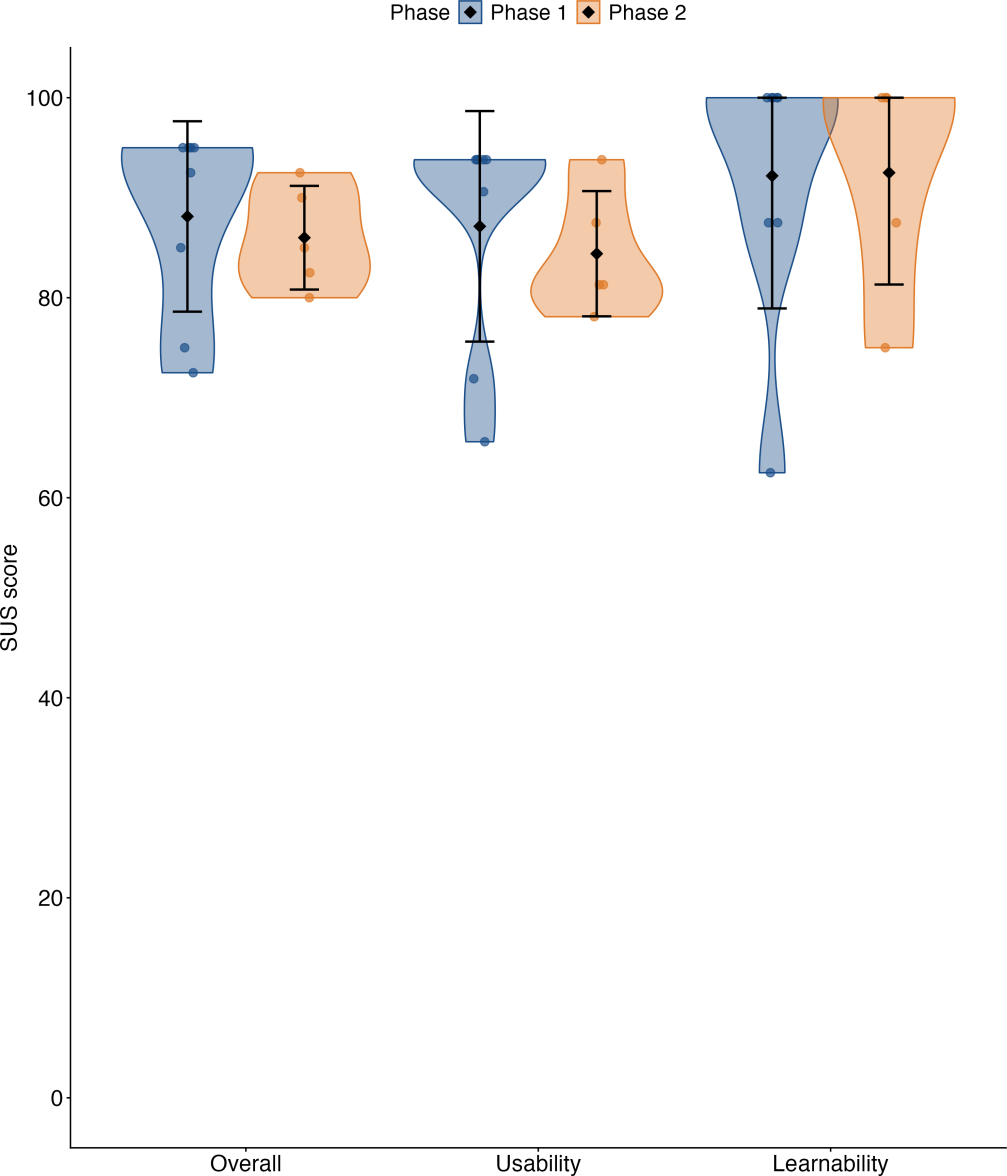
System Usability Scale overall and subscale scores across evaluation phases. Diamonds represent means and error bars represent ±1 SD, capped at the System Usability Scale maximum of 100. Individual data points are shown as circles. SUS: System Usability Scale.

All SUS scores were considered excellent. After the first evaluation phase, the overall mean usability score from the SUS was 88.12 (SD 9.52), median 92.50 (range 72.5‐95.0), and mean scores for the usability and learnability subscales were 87.14 (SD 11.53), median 93.80 (range 65.6‐93.8) and 92.19 (SD 13.26), median 100.0 (range 62.5‐100.0). After the second evaluation phase, the overall mean usability score was 86.00 (SD 5.18), median 85.00 (range 80.0‐92.5), and mean scores for the usability and learnability subscales were 84.40 (SD 6.26), median 81.30 (range 78.1‐93.8) and 92.50 (SD 11.18), median 100.0 (range 75.0‐100.0), respectively (Figure 3). Although overall and usability subscale scores showed a slight decrease from phase 1 to phase 2, both phases remained in the excellent range. Learnability subscale scores were largely unchanged across phases, suggesting that the app remained highly intuitive even as the prototype increased in complexity from a single learning objective to a full three-objective module.

## Discussion

This study described the iterative design process and presented the usability testing of the REPS mobile app. REPS is a cognitive behavioral therapy and REBT-based mental fitness tool developed for newly enlisted US Navy sailors using a human-centered design approach. Across two evaluation phases with 13 first-term sailors, qualitative findings highlighted strong perceived relevance and cultural fit, identified areas for functional and esthetic refinement, and surfaced actionable recommendations that directly shaped app improvements. Quantitatively, SUS scores fell in the excellent-to-best-imaginable range across both phases, supporting the app’s usability and learnability. Together, these findings suggest that REPS is a promising, scalable digital tool for supporting mental fitness and universal suicide prevention among newly enlisted service members.

A consistent theme across both evaluation phases was that sailors found the REPS app relatable and grounded in their lived experiences. Sailors highlighted the cultural relevance of the scenario-based activities and the app’s alignment with the specific stressors of naval life, including deployment-related social isolation and the unpredictability of operational schedules. This finding is theoretically meaningful. Prior systematic review research has identified a lack of perceived fit as one of the most commonly cited barriers to engagement with DMHIs [18]. Findings suggest that the HCD process aided in building perceived fit into the REPS app from the outset of app creation.

This finding aligns with broader HCD literature emphasizing co-design as a mechanism for enhancing app uptake. Lyon et al [17] argued that user-centered approaches increase engagement because they produce tools better equipped to meet the needs and interests of the intended population. Our results are consistent with this argument by involving sailors in the design process. This process helped build app content that resonated with sailors in ways that purely top-down approaches may have struggled to achieve [14]. This is particularly relevant for military populations, where concerns about stigma create additional barriers to engagement with care [7,8]. The ability to use REPS privately and in a culturally familiar context may help to address engagement issues that are common with DMHIs.

Both evaluation phases produced SUS scores in the excellent range, comparing favorably with published SUS benchmarks for DMHIs [41]. Notably, learnability subscale scores were particularly high across both phases, suggesting that sailors could orient to the app’s structure and navigation quickly and with minimal instruction. This is an important finding for a population characterized by limited free time, high operational demands, and deployment contexts that may preclude access to technical support. The slight decrease in overall and usability subscale scores between phases is worth noting. This is likely attributable to the increased complexity of the phase 2 prototype, which expanded from a single learning objective to a complete three-objective module, rather than reflecting a decline in app quality. That scores remained in the excellent range despite this increased complexity suggests the iterative design refinements were effective in maintaining usability as content depth grew.

For nearly 20 years, the US DoD and the Department of VA have led the way in applying HCD principles to develop digital mental health tools for service members and veterans. Key apps like PTSD Coach (US Department of VA and the US DoD)**,** Mindfulness Coach (US Department of VA), and Virtual Hope Box (US Department of VA and the US DoD) were developed through iterative, user-informed processes to enhance accessibility, usability, and clinical effectiveness [42-44]. While these tools have expanded access to evidence-based psychological support, most function as stand-alone resources or treatment add-ons focused on specific clinical conditions. Building on this foundation**,** the REPS app extends the use of HCD to design and evaluate a comprehensive, training-focused mobile platform organized into sequential learning modules. Each module offers skill-building content designed to improve overall mental fitness, including emotion regulation, cognitive flexibility, and problem-solving, rather than focusing solely on symptom management. Developed closely with active-duty sailors**,** REPS addresses the realities of naval life, such as long deployments, social isolation, and high workload demands. This structured, performance-oriented design positions REPS as a next-generation tool to bolster resilience and psychological readiness among service members.

User feedback across both evaluation phases directly informed meaningful design changes, illustrating the iterative HCD process. Two findings are worth noting. First, no sailor spontaneously engaged with clickable headers, highlighted keywords, or knowledge nuggets in the prototype. Similarly, sailors consistently navigated to an incorrect icon when searching for the badge feature, and no sailor clicked the “Play Again” option in the choose-your-own-adventure activity despite expressing interest in exploring alternative response pathways. These findings suggest that engagement features may need to be actively noticeable through animation or prompts rather than embedded passively in the interface. Second, several sailors expressed unfamiliarity with psychological terminology such as “maladaptive,” despite finding the content broadly relatable. The REPS team used this feedback to refine the app by defining key terms in accessible language prior to their use in activities and incorporating concrete and relatable examples. Using HCD principles to highlight interactive terms and define psychological terminology helped to refine the user experience between the two phases.

This study has numerous limitations. First, the sample size is small and may not generalize to the broader population of Navy sailors or other service members. However, the small sample size in this study is appropriate for usability studies and reflective of other HCD projects [45]. Second, sailors were stationed at a single ship and may have had characteristics that influenced their willingness to participate in the evaluation phases. Third, although both usability and acceptability were tested, objective engagement metrics were not collected during these prototype evaluation phases. Fourth, participant race and ethnicity were not collected. This limits the representativeness of the sample. Fifth, the qualitative information was coded by a single coder, increasing the risk of coder bias. Sixth, usability was assessed using the SUS alone. Future studies should consider supplementing the SUS with broader user experience measures, such as the User Experience Questionnaire, to more comprehensively assess dimensions of user experience. Seventh, because sailor recruitment was facilitated by commanding officers, sailors may have felt social pressure to participate and to respond favorably, potentially leading to an overestimation of the app’s usability and acceptability.

This study contributes to the growing literature on HCD-developed DMHIs for active-duty service members. REPS is the first mobile app designed to use mental fitness as a universal suicide prevention approach for newly enlisted Navy sailors, which is a population at elevated suicide risk. The excellent usability and learnability scores, combined with strong qualitative support for cultural relevance and perceived fit, provide a promising foundation for the app’s continued development and evaluation. As the REPS app moves toward full deployment with four complete modules, future research should examine its effectiveness in improving mental fitness and reducing suicide risk. More broadly, the REPS development process offers a replicable model for applying HCD principles to build mental fitness tools that are both evidence-based and meaningfully shaped by the communities they are designed to serve.

## Supplementary material

10.2196/89994 Multimedia Appendix 1 Interview transcript for phase 1.

10.2196/89994 Multimedia Appendix 2 Interview transcript for phase 2.

## Abbreviations

DMHI: digital mental health intervention
DoD: Department of Defense
HCD: human-centered design
PTSD: posttraumatic stress disorder
REBT: Rational Emotive Behavior Therapy
REPS: Rational Thinking, Emotion Regulation, and Problem Solving
SUS: System Usability Scale
VA: Veterans Affairs
