# Supplementary material for: Development of the Rational Thinking, Emotion Regulation, and Problem-Solving Mental Fitness Mobile App for US Navy Sailors: Qualitative and Quantitative Usability Evaluation
Source: JMIR Form Res. 2026 Jul 31;10:e89994. doi: 10.2196/89994 (PMC13427060; doi:10.2196/89994)
Supplement: Multimedia Appendix 1 [file formative-v10-e89994-s001.docx]

**PARTICIPANT #: ___**

**Welcome (2min)**

**Script for UX Coordinator**

Welcome, and thank you for volunteering to participate in this evaluation. Your participation will help us better understand how a mobile application could benefit sailors' mental fitness.

*To explore this, our team will ask you some questions and run you through a mobile app idea to get your feedback. This session will take 30-40 minutes in total.*

*Your participation and honest feedback are appreciated, so please speak freely and openly.*

*Do you have any questions before I hand you over to [UX Specialist Name]?*

**CONSENT TO RECORD (1min)**

*Before we begin, is it OK to audio record our session? The recording will not be shared outside the study team. The recordings will help me ensure I am accurate in my reporting.*

**INTERVIEW QUESTIONS: (5 - 8 minutes)**

OK, I have a few questions to start us off.

1. What do you do in the Navy? What is your job?
   - Is your job fun? What do you like about it?
   - Is it stressful? How so?
2. So, when you hear the term “mental fitness,” what comes to mind?
   - How would you define it?
   - If they do not know, provide definition:
     1. **Mental fitness means keeping our brains and emotional health in top shape. It helps us cope with stress and manage life challenges so that we can continue to thrive in our personal and professional lives.**
3. How important is mental fitness for Navy sailors and their careers?
   - How so?/Why is that?
4. Based on your experience, what kinds of life challenges or stressful situations do new sailors experience?
   - Can you give an example of something that happened to you or someone you know?
   - Examples:
     - Such as leaving home for the first (to join the Navy)
     - Extended deployments
     - Relationship troubles
     - Challenges with authority
     - Feeling isolated/lonely
     - Other
5. **MULTIPLE CHOICE:**

There are lots of strategies to help our mental fitness. If any, which of the following strategies or tools do you use to support your mental fitness? (Check all that apply)

- - - Practice Mindfulness/Meditation
    - Journaling
    - Fitness/Exercise
    - Connect with family or friends
    - Practice positive thinking/gratitude
    - Actively work on my communication skills (e.g., listening more, being present, thinking before speaking)
    - Learn a new skill
    - Other (please explain)

1. Do you use web or mobile applications to support your mental fitness?
   - If so, which ones?
   - Examples: Meditation/Mindfulness, mood tracking, exercise/fitness, learning apps

**Mobile App Tasks: 15 minutes**

This mobile app concept is intended to help sailors learn and practice various strategies to strengthen their mental fitness. As we review this prototype, I will ask you to think aloud about what you see and experience on each screen. Just to get your first impressions. I will also ask for your thoughts along the way.

| **Task** | | **Instruction/Probes** | **Pass/Fail** |
| --- | --- | --- | --- |
| **1** | **Home Screen** | First thing – what do you notice about the home page? |  |
| **2** | **Start a Module** | You want to start your first lesson. Where would you go? |  |
| **3** | **Recognizing Change Activity** | **Start screen:** How would you complete this activity?  **Results screen:** What does this screen show you? |  |
| **4** | **Identifying Types of Thoughts** | **Start screen:** How would you complete this activity?  **Results screen:** What does this screen show you?  *[Moderator: If they get the activity “correct”, go back and ask them to put some statements in the wrong bucket. ]* |  |
| **5** | **Texting Activity** | Read through the screen. Without clicking on anything, which response would you choose?  **Select Top Answer:** OK, we have set it up for you to click the first response for this prototype. Go ahead and do that. |  |
| **6** | **Footer Icons** | For the icons below, what do you think these icons mean?  If you could click on them, where would they take you? |  |
| **7** | **Practice Your REPS screen** | *[Moderator: Have participant select the middle icon]*  What would you expect you to do in this section of the app?  *[Moderator: If they do not know, tell them the intent of this section.]*  What do you think about this idea? |  |

**Debrief questions: (5-8 minutes)**

Thanks for going through the prototype. I have some questions about your experience going through it.

1. What did you like about the mobile app concept?
2. What did you dislike or think could be improved?
3. If you had a magic wand, what would you like to see for a mental fitness app for Navy sailors?

**End of Session**

Thank you for participating today. We’re finished with the app review.

Please return to [UX Coordinator Name]. They will ask you to complete a brief questionnaire about your experience using the REPS mobile app. It should only take a few minutes.
