# Supplementary material for: Development of the Rational Thinking, Emotion Regulation, and Problem-Solving Mental Fitness Mobile App for US Navy Sailors: Qualitative and Quantitative Usability Evaluation
Source: JMIR Form Res. 2026 Jul 31;10:e89994. doi: 10.2196/89994 (PMC13427060; doi:10.2196/89994)
Supplement: Multimedia Appendix 2 [file formative-v10-e89994-s002.docx]

**Participant #_________**

**Welcome**

*Thank you for participating in this usability test. Today, we are evaluating a mobile app focused on mental fitness training. Your feedback will be invaluable in helping us improve the overall app and tailor the experience to better suit Navy sailors.*

*I want to emphasize that I am not evaluating you; I am evaluating the mobile app.*

*We are looking to understand if:*

- *the content is understandable and clear,*
- *if the app is easy to navigate,*
- *and if anything is confusing or out of place.*

*We also want your thoughts or ideas on improving the app experience.*

*For this review, we will walk through a learning module that consists of 3 learning objectives—or “mini-lessons.” I will provide a few scenarios as we go and ask you to complete some tasks.*

*As you use the app, I will observe and take notes. Please be free to ask any questions, comments/observations along the way. I am all ears and want to know what you’re thinking.*

*Also, there is no need to rush through the tasks; take your time. Remember, I am not testing you—I am testing the app.*

*Do you have any questions before I hand you over to [UX Specialist Name]?*

**Main Scenario:**

Your commander has asked you to complete a new mental fitness training course. She provides you with the information to download onto your phone.

| **Scenario:**  *You want to understand this app's purpose. Read through the first few screens and then stop once you land on the home screen.* | | |
| --- | --- | --- |
| **Screens/Features** | **P/F** | **Notes** |
| **Onboarding Screens** |  |  |
| ---END TASK--- | | |

| **Question for participant** | **Notes:** |
| --- | --- |
| 1. Based on what you just read, what do you think this app is about? |  |

# **Objective 1: Change is Constant**

| **Scenario:**  *You are ready to start the training. Where would you go first?*  *Go ahead and complete the first learning objective.* | | | |
| --- | --- | --- | --- |
| **SCREENS - Trivia Questions** | | |  |
| **Trivia 1**   1. Selected an answer. 2. On first try, user selected another answer if they got it wrong. | P/F |  |  |
|  | P/F |  |  |
| **Trivia 2**   1. Selected an answer. 2. On first try, user selected another answer if they got it wrong. | P/F |  |  |
|  | P/F |  |  |
| SCREENS - **Adaptive vs. Maladaptive responses** | | |  |
| Clicked on the word: Adaptive | P/F |  |  |
| Clicked on the word: Maladaptive | P/F |  |  |
| SCREENS - **Click to Reveal** | | |  |
| Clicked on both cards before trying to advance. | P/F |  |  |
| SCREENS - **Reinforce** | | |  |
| 1. Selected an answer. 2. In first try, user selected another answer if they got it wrong. | P/F |  |  |
|  | P/F |  |  |
| ---End Task – (wait to go to the Home screen until after you ask questions). | | |  |

| **Questions for participant** | **Notes:** |
| --- | --- |
| 1. What stood out to you in this first learning objective? |  |
| 1. Anything you liked about the objective? Disliked? |  |
| 1. Did you encounter anything that was confusing or unclear? |  |

### **Objective 2: Adaptive thinking**

| **Scenario:**  *After taking a break, you came back to check out another learning objective.*  *Go ahead and complete Objective 2.* | | |
| --- | --- | --- |
| **SCREENS – Select the best answer (Quiz)** | | |
| **Which is a thought 1?**   1. Selected an answer. 2. User selected another answer if they got it wrong. | P/F |  |
| **Which is a thought 2?**   1. Selected an answer. 2. User selected another answer if they got it wrong. | P/F |  |
|  | P/F |  |
| **Which is an emotion 1?**   1. Selected an answer. 2. User selected another answer if they got it wrong. | P/F |  |
|  | P/F |  |
| **Which is an emotion 2?**   1. Selected an answer. 2. User selected another answer if they got it wrong. | P/F |  |
|  | P/F |  |
| **Which is an action 1?**   1. Selected an answer. 2. User selected another answer if they got it wrong. | P/F |  |
|  | P/F |  |
| **Which is an action 2?**   1. Selected an answer. 2. User selected another answer if they got it wrong. | P/F |  |
|  | P/F |  |
| **Which is a physical sensation 1?**   1. Selected an answer. 2. User selected another answer if they got it wrong. | P/F |  |
|  | P/F |  |
| **Which is a physical sensation 1?**   1. Selected an answer. 2. User selected another answer if they got it wrong. | P/F |  |
|  | P/F |  |
|  |  |  |
| SCREENS – **It’s all connected** | | |
| **Broken phone 1**  Flipped triangle. | P/F |  |
| **Broken phone 2**  Flipped triangle. | P/F |  |
| **Got a promotion 1**  Flipped triangle. | P/F |  |
| **Got a promotion 2**  Flipped triangle. | P/F |  |
| SCREENS - **Reinforce** | | |
| **I know my Chief thinks I’m worthless.**   1. Selected an answer. 2. User selected another answer if they got it wrong. | P/F |  |
|  | P/F |  |
| **Jealousy**   1. Selected an answer. 2. User selected another answer if they got it wrong. | P/F |  |
|  | P/F |  |
| **Exhaustion**   1. Selected an answer. 2. User selected another answer if they got it wrong. | P/F |  |
|  | P/F |  |
| **Asks a shipmate how they’re doing**   1. Selected an answer. 2. User selected another answer if they got it wrong. | P/F |  |
|  | P/F |  |
| ---End Task – Go to HOME SCREEN --- | | |

| **Questions for participant** | **Notes:** |
| --- | --- |
| 1. What stood out to you in this second learning objective? |  |
| 1. Anything you liked about the objective? Disliked? |  |
| 1. Did you encounter anything that was confusing or unclear? |  |

## **Objective 3: Thinking Traps**

| **Note to Participant:**  *OK, we are on the last objective. As you go through it, I will ask you to stop at one point because I will need to clarify a feature you will see.*  *Scenario:*  *Go ahead and complete learning objective 3.* | | | |
| --- | --- | --- | --- |
| **SCREENS – Introducing the ABCs** | | |  |
| **Knowledge Nugget**  Clicked on Knowledge Nugget | P/F |  |  |
| SCREENS – **Practicing the ABCs** | | |  |
| Consequences – Thoughts |  |  |  |
| **Play again**  After first time through activity --Clicked on “play again” | P/F |  |  |
| **Play again (x2)**  After second time through activity -- Clicked on “play again”  *(Note to moderator -- Does the user think there are more activities to go through since the Play Again button is still there?)* | P/F |  |  |
| SCREENS - **Reinforce** | | |  |
| 1. Selected an answer. 2. User selected another answer if they got it wrong. | P/F |  |  |
|  | P/F |  |  |
| ---End Task – Go to HOME SCREEN --- | | |  |

| **Questions for participant** | **Notes:** |
| --- | --- |
| 1. What stood out to you in this last learning objective? |  |
| 1. Anything you liked about the objective? Disliked? |  |
| 1. Did you encounter anything that was confusing or unclear? |  |

## **Awards/Medals**

| **Task:**  *You want to see the medals/achievements you collected during the training. Where would you go?* | | | |
| --- | --- | --- | --- |
| **SCREENS – Earn medals/achievements** | | **Notes** | |
| 1. Found the screen on first try. | P/F |  | |
| **Question for participant** | **Notes:** | |  |
| 1. What do you think about the idea of collecting medals/awards? |  | |  |

## **Do Your REPS**

| **Task:**  *You want to practice some of the activities from the training. Where would you go?* | | | |
| --- | --- | --- | --- |
| **SCREENS – Earn medals/achievements** | **Notes** | | |
| 1. Found the screen on first try. | | P/F |  |
| **Question for participant** | | **Notes:** | |
| 1. What do you think about the idea practicing new versions of the activities you saw in the learning objectives? | |  | |

Thanks for going through the prototype. I have some questions about your experience going through it.

1. What did you like about the mobile app concept?
2. What did you dislike or think could be improved?
3. If you had a magic wand, what would you like to see for a mental fitness app for Navy sailors?

**End of Session**

Thank you for participating today. We’re finished with the app review.

Please return to [UX Coordinator Name]. They will ask you to complete a brief questionnaire about your experience using the REPS mobile app. It should only take a few minutes.
